# Concomitant Liwen procedure and minimally invasive direct coronary artery bypass via a single access: a first-in-human case report

**DOI:** 10.1093/ehjcr/ytag312

**Published:** 2026-06-01

**Authors:** Xinyu Li, Shurui Xie, Zhengchun Yu

**Affiliations:** Department of Ultrasound, Wuhan Asia Heart Hospital Affiliated to Wuhan University of Science and Technology, Wuhan Clinical Medical Research Center of Cardiovascular Imaging, No. 753 Jinghan Avenue, Wuhan 430022, China; Department of Medicine, School of Medicine, Wuhan University of Science and Technology, No. 947 Heping Avenue, Qingshan District, Wuhan 430065, China; Department of Ultrasound, Wuhan Asia Heart Hospital Affiliated to Wuhan University of Science and Technology, Wuhan Clinical Medical Research Center of Cardiovascular Imaging, No. 753 Jinghan Avenue, Wuhan 430022, China; Department of Ultrasound, Wuhan Asia Heart Hospital Affiliated to Wuhan University of Science and Technology, Wuhan Clinical Medical Research Center of Cardiovascular Imaging, No. 753 Jinghan Avenue, Wuhan 430022, China

**Keywords:** Hypertrophic obstructive cardiomyopathy, Minimally invasive direct coronary artery bypass, Myocardial contrast-enhanced echocardiography, Case report

## Abstract

**Background:**

Advances in anaesthesia and minimally invasive surgical techniques have expanded therapeutic options for patients with complex cardiac disease. While conventional open-heart surgery carries significant risks for this population, hybrid strategies offer a viable alternative.

**Case summary:**

We report the first case of concurrent hypertrophic obstructive cardiomyopathy (HOCM) and coronary artery disease (CAD) treated with a one-stage hybrid strategy: combining the Liwen procedure (echocardiography-guided percutaneous intramyocardial septal radiofrequency ablation) with minimally invasive direct coronary artery bypass (MIDCAB) via a single access. This approach effectively addressed both left ventricular outflow tract obstruction and myocardial ischaemia, while reducing trauma and accelerating recovery.

**Discussion:**

This case demonstrates that the concomitant Liwen procedure and MIDCAB through a single minimally invasive access is a feasible and promising strategy for patients with coexisting HOCM and CAD, offering reduced trauma and faster recovery compared to traditional approaches.

Learning points

- This first-in-human case demonstrates the feasibility of a single-access, one-stage hybrid approach combining Liwen procedure and off-pump minimally invasive direct coronary artery bypass to treat concomitant hypertrophic obstructive cardiomyopathy and coronary artery disease, avoiding sternotomy and bypass.
- This strategy reduces trauma and accelerates recovery by integrating echo-guided septal ablation with beating-heart bypass, offering a valuable alternative for high-risk patients with complex cardiac disease.

## Introduction

Conventional open-heart surgery carries substantial risks, particularly for patients with impaired cardiac function and multiple comorbidities.^1^ Hybrid strategies that integrate interventional and surgical techniques—such as combining the Liwen procedure with off-pump minimally invasive direct coronary artery bypass (MIDCAB)—aim to minimize trauma and maximize procedural efficiency. This report presents the first case of a one-stage Liwen procedure and MIDCAB performed through a single minimally invasive access in a 68-year-old woman with severe hypertrophic obstructive cardiomyopathy (HOCM) and coronary artery disease (CAD). The case demonstrates the technical feasibility, synergistic advantages, and clinical potential of this combined strategy for such patients.

## Summary figure

**Figure:**
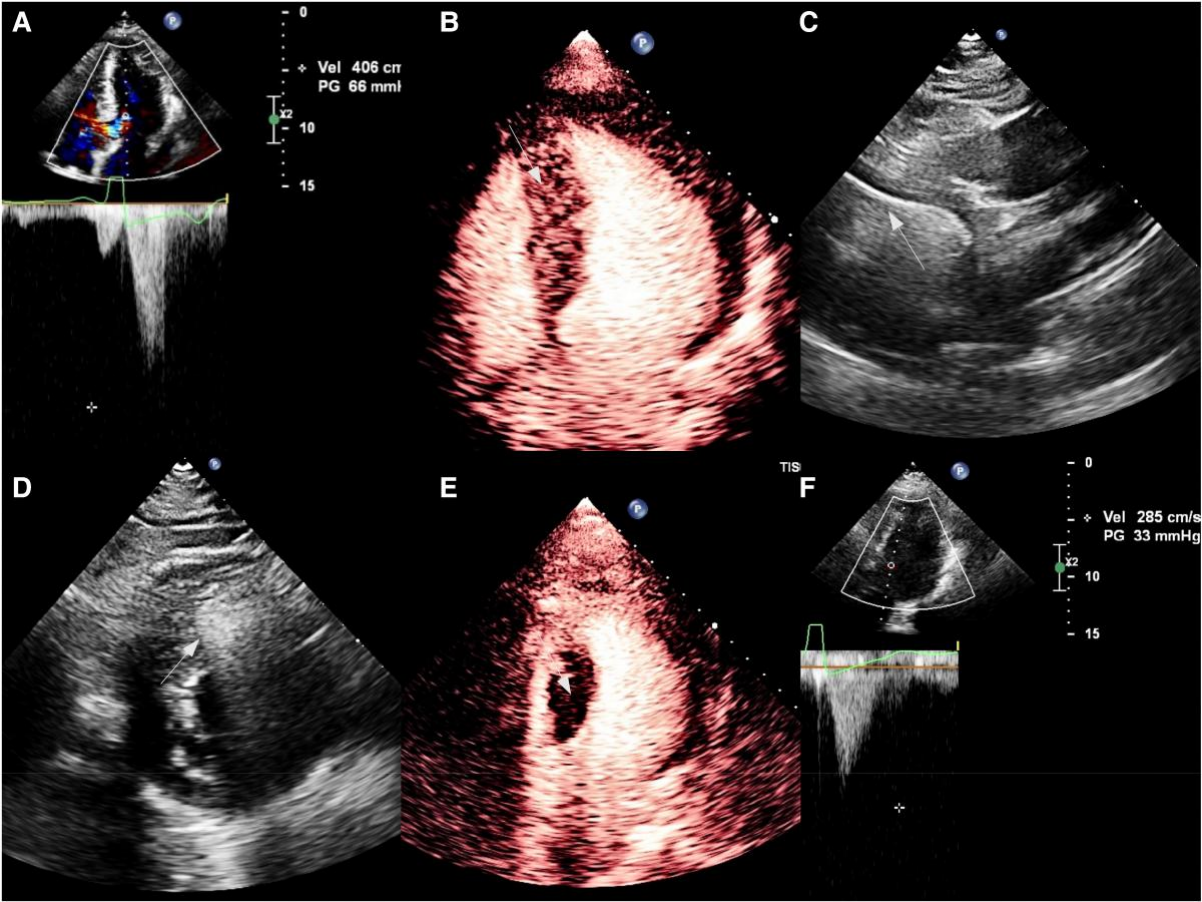


## Case presentation

**Figure 1:**
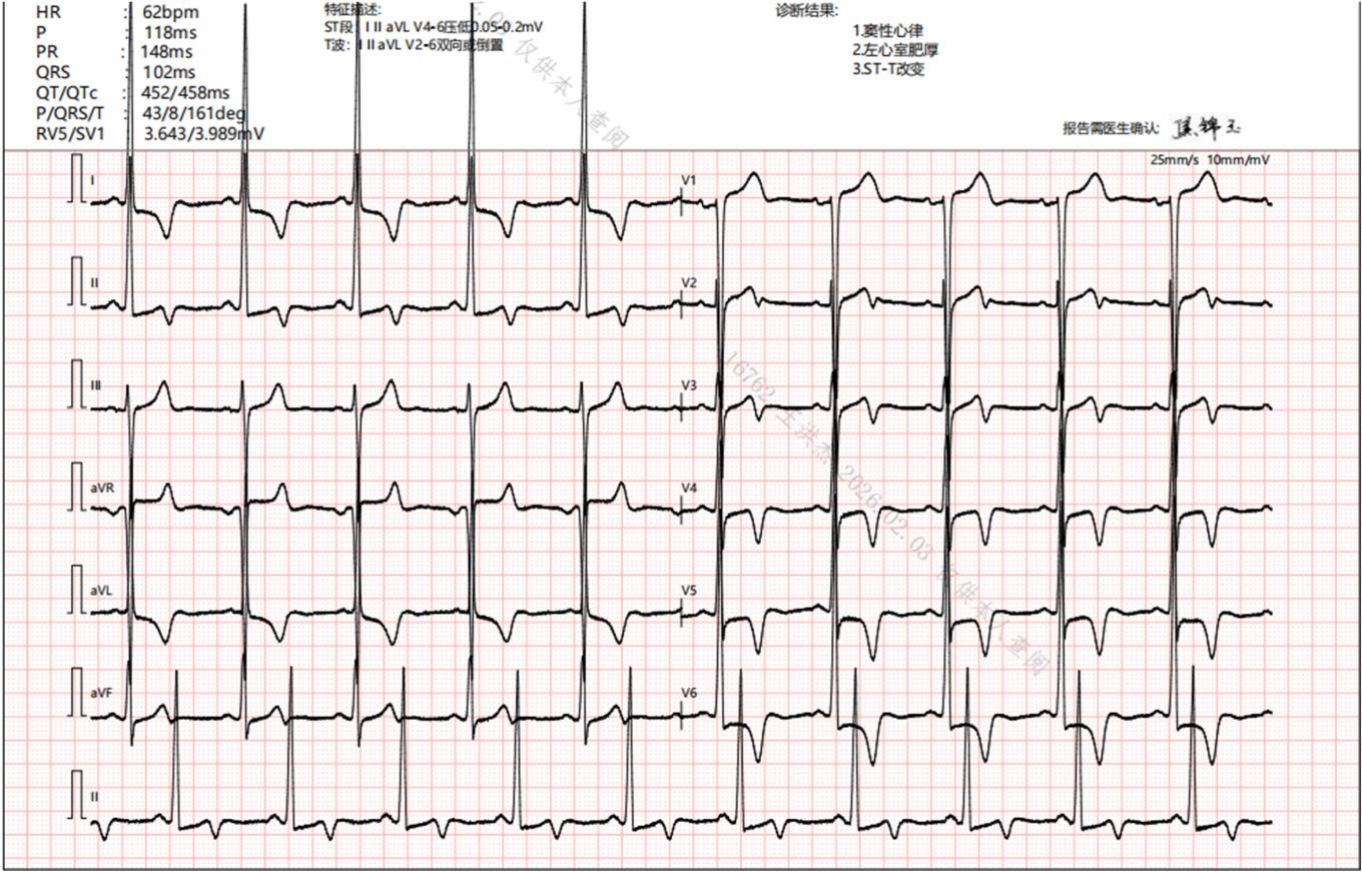
Pre-operative electrocardiogram. The tracing suggests left ventricular hypertrophy with ST-T segment changes.

**Figure 2:**
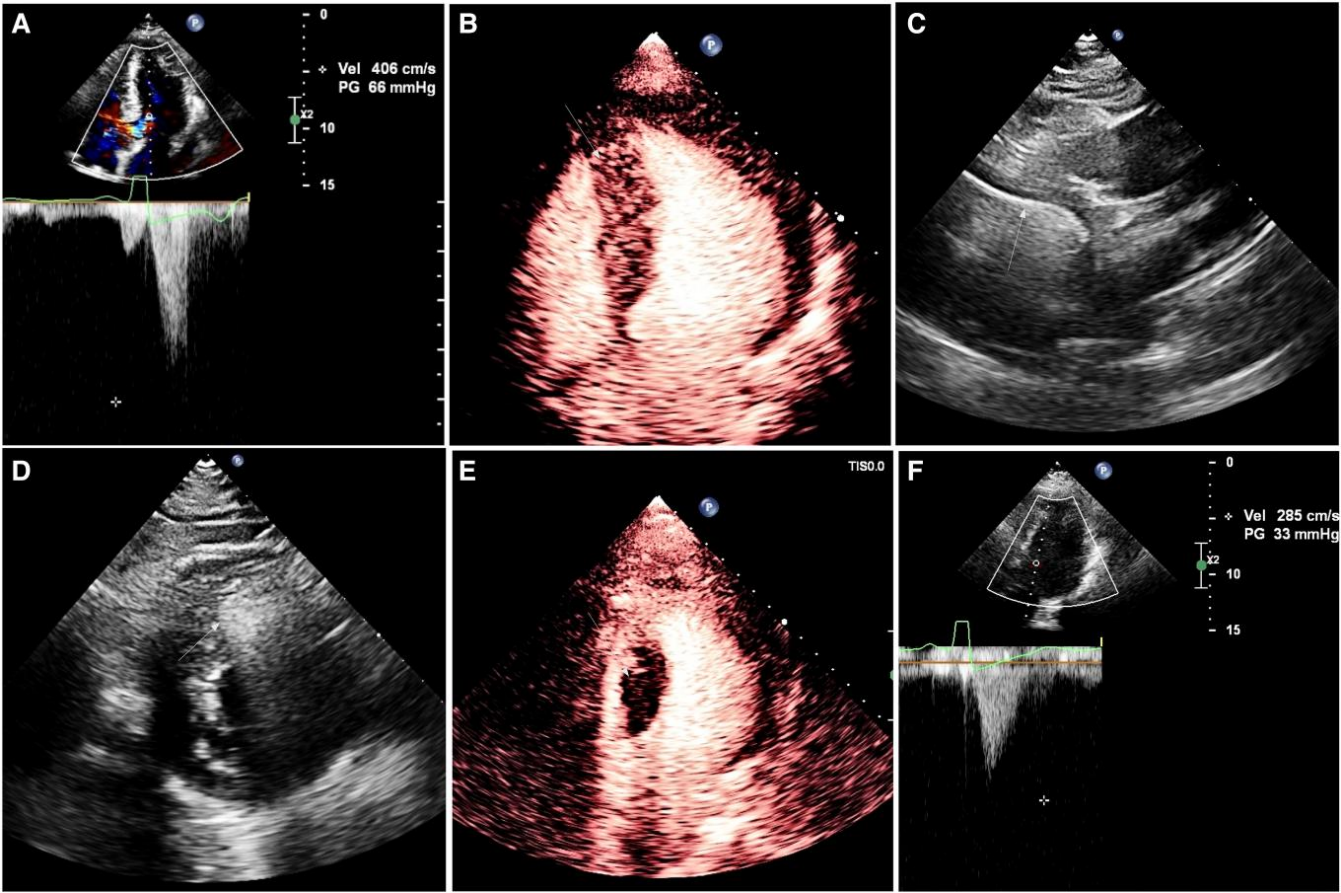
Imaging examinations before, during, and after surgery. (*A*) Pre-operative transthoracic echocardiography demonstrating left ventricular outflow tract obstruction. (*B*) Myocardial contrast-enhanced echocardiography showing severe septal hypertrophy (thickness 22 mm). (*C*) The position of the radiofrequency ablation needle within the basal anterior septum was confirmed utilizing multiple echocardiographic views. (*D*) Arrow indicates the hyperechoic gas-bubble zone following radiofrequency ablation. (*E*) Post-ablation myocardial contrast-enhanced echocardiography revealing a contrast filling defect within the ablation zone. (*F*) Post-operative transthoracic echocardiography demonstrating a left ventricular outflow tract gradient of 33 mmHg.

A 68-year-old female with a history of hypertension, CAD, interstitial pulmonary oedema, emphysema, and no prior cardiac surgery presented with New York Heart Association Class III exertional chest tightness and dizziness (relieved by rest). Physical examination revealed a Grade 3/6 systolic murmur at the left parasternal third–fourth intercostal spaces. The electrocardiogram showed left ventricular hypertrophy with ST-T changes (*Figure 1*). Transthoracic echocardiography (TTE) demonstrated severe asymmetric septal hypertrophy (septal 22 mm, posterior wall 17 mm), left ventricular outflow tract (LVOT) obstruction with peak velocity of 4.1 m/s and pressure gradient of 66 mmHg (*Figure 2A*), and positive systolic anterior motion of the mitral valve (SAM) with anterior leaflet thickening. Myocardial contrast-enhanced echocardiography (MCE) findings were consistent with the TTE results (*Figure 2B*; Supplementary material online, *Video S1*). Coronary angiography demonstrated 80%–90% stenosis in the proximal left anterior descending artery (LAD). Given the patient’s age (68 years), comorbidities (pulmonary oedema, emphysema), impaired cardiopulmonary reserve (second-grade 6-min walk test)—11.2% predicted in-hospital mortality for conventional cardiac surgery (EuroSCORE Ⅱ),^2^ and concurrent severe myocardial ischaemia, LVOT obstruction, and surgically indicated structural heart disease (SAM with anterior leaflet thickening), the multidisciplinary heart team recommended a one-stage minimally invasive hybrids surgery after comprehensive discussion.

Under ultrasound guidance, a Cool-Tip ACT-1520 needle was percutaneously advanced along the long axis of the interventricular septum from the apex to the basal anterior septum (*Figure 2C*; Supplementary material online, *Video S2*). Radiofrequency ablation was initiated with incremental energy delivery; real-time imaging demonstrated an expanding hyperechoic gas-bubble zone originating from the needle tip, indicating effective ablation (*Figure 2D*; Supplementary material online, *Video S3*). Subsequently, under ultrasound guidance, the needle was sequentially withdrawn and its trajectory adjusted to perform targeted ablation at the following sites: the basal anterior-septal junction, the mid anterior-septal junction, the basal posterior septum, the mid-posterior septum, and the apical segment. Following completion of the Liwen procedure, MCE confirmed a contrast filling defect in the septal ablation zone (*Figure 2E*; Supplementary material online, *Video S4*). Minimally invasive direct coronary artery bypass was then performed through an 8-cm left fifth intercostal incision: the great saphenous vein was harvested and anastomosed end-to-side to the mid-proximal LAD on the beating heart. Post-operative TTE showed significant improvement in LVOT obstruction (peak velocity 2.9 m/s, pressure gradient 33 mmHg) and ventricular function (*Figure 2F*). The patient had an uneventful recovery, with 5 days in the ICU and discharge on post-operative Day 9 (total hospital stay 17 days). At the 1-month post-operative follow-up, TTE confirmed a significant reduction in the LVOT gradient to 9 mmHg (peak velocity 1.5 m/s).

## Discussion

This case represents the first report of simultaneous Liwen procedure and MIDCAB via a single minimally invasive access, offering a one-stage solution for both HOCM and CAD. Compared with conventional staged procedures, this strategy eliminates the need for multiple interventions and resolves dilemmas in antiplatelet management. Compared with standard sternotomy, it markedly reduces surgical trauma and complication rates, accelerates post-operative recovery, and is particularly suitable for patients with impaired cardiac function and multiple comorbidities.^1^ In the presence of hypertrophic cardiomyopathy complicated by significant CAD (notably proximal LAD stenosis), coronary artery bypass grafting is typically recommended over percutaneous coronary intervention.^3^ A saphenous vein graft was chosen over the internal mammary artery, considering the patient’s severely impaired cardiopulmonary reserve secondary to severe emphysema, pulmonary oedema, and 6-min walk test Grade 2. This choice minimized surgical trauma, maximized procedural efficiency, and prioritized peri-operative safety.

This hybrid strategy combining the Liwen procedure and MIDCAB via a single minimally invasive access offers three key advantages. First, the minimally invasive incision avoids extensive tissue trauma associated with open-heart surgery, reducing intra-operative bleeding and injury risks. Second, the Liwen procedure—an innovative interventional technique—enables precise radiofrequency ablation of obstructive myocardial tissue under real-time ultrasound guidance, effectively relieving LVOT obstruction and improving cardiac function^4^; MIDCAB further provides low peri-operative morbidity/mortality, excellent long-term survival, freedom from major adverse events and angina, and minimal trauma.^5^ Third, MIDCAB avoids cardiopulmonary bypass-related injuries, mitigates myocardial ischaemia-reperfusion damage, and shortens operative time.

## Conclusion

The combined Liwen procedure and MIDCAB is a safe, effective, and innovative treatment for concomitant HOCM and CAD. This single-stage hybrid approach via a single minimally invasive access reduces trauma, avoids bypass, and accelerates recovery, offering a valuable alternative to traditional surgeries.

## Supplementary Material

ytag312_Supplementary_Data

## Data Availability

The data underlying this article are available in the article and in its online Supplementary material.

## Lead author biography

My name is Li Xinyu. I am 24 years old and currently a medical post-graduate student at Wuhan University of Science and Technology. I am undergoing standardized training at Wuhan Asia Heart Hospital, located in Wuhan City, Hubei Province.

## Notes

**Consent:** The authors confirm that written consent for submission and publication of this case report including images and associated text has been obtained from the patient, in line with COPE guidance.
